# Editorial: Passive brain-computer interfaces: moving from lab to real-world application

**DOI:** 10.3389/fncom.2026.1826791

**Published:** 2026-03-25

**Authors:** Vincenzo Ronca, Luca Longo, Rossella Capotorto, Pietro Aricò

**Affiliations:** 1 Department of Computer, Control, and Management Engineering “Antonio Ruberti”, Sapienza University of Rome, Rome, Italy; 2 The Artificial Intelligence and Cognitive Load Research Lab, University College Cork, Cork, Ireland; 3 Department of Anatomical, Histological, Forensic and Orthopedic Sciences, Sapienza University of Rome, Rome, Italy

**Keywords:** Brain Computer Interface (BCI), EEG, human factor (HF), neurophysiology, real word applications

Passive brain–computer interfaces (pBCIs) have progressively evolved from proof-of-concept laboratory systems to increasingly realistic candidates for real-world deployment (Aricò et al., 2018; Aricó et al., 2017a; Belo et al., 2021; Mahmoudi et al., 2025; Beauchemin et al., 2024; Zhou et al., 2017). Unlike active BCIs, which rely on intentional control signals, pBCIs aim to decode spontaneous or task-embedded brain activity to infer users' cognitive, affective, or physiological states. This paradigm shift opens the door to continuous, unobtrusive monitoring of mental states such as stress, workload, engagement, fatigue, and motor intention, with applications spanning healthcare, education, human–machine interaction, and safety-critical environments (Borghini et al., 2020; Ronca et al., 2025a, 2024a, 2023, 2025b; Di Flumeri et al., 2019, 2022; Aricò et al., 2019; Aricó et al., 2017b; Chi et al., 2012).

Yet, the transition from laboratory validation to real-world application remains challenging. EEG signals are inherently noisy, non-stationary, and subject-specific (Ronca et al., 2024b; Anders et al., 2020; Kobler et al., 2020). Experimental paradigms often confound cognitive states with task structure. Wearable hardware must balance signal quality with comfort and usability (Alberto et al., 2018; Vincenzo et al., 2025). Moreover, as deep learning methods dominate decoding pipelines, interpretability and neuroscientific plausibility become central concerns (Mashhadi et al., 2020; Ozdemir et al., 2022; Zhang et al., 2021).

The contributions gathered in this Research Topic address these challenges from complementary perspectives, collectively advancing the methodological, technological, and conceptual foundations required to bring passive BCIs into everyday settings.

A first critical axis concerns advanced modeling of complex EEG dynamics. The study by Wang and Wang introduces a multi-branch deep learning framework for fine-grained motor imagery classification. By integrating graph attention networks (GAT), recurrent units, Transformers, and frequency-specific convolutional layers, their architecture explicitly models the spatial, temporal, and spectral structure of EEG signals. Importantly, this work goes beyond performance gains by embedding biologically informed priors, such as phase locking value (PLV)–derived connectivity, into the model structure, and by leveraging SHAP analyses to identify neurophysiologically meaningful channels and frequency bands.

Additionally, concerns ecological validity and the decoding of brain activity in naturalistic contexts were approached by Alasfour and Gilja by investigating whether stable, consistent neural signatures can discriminate real-world behavioral states. Using long-duration human ECoG recordings with coarse video-based annotations, they show that discriminative spectro-spatial features, spanning θ/α and low/high γ activity across sensorimotor and temporal regions, can be identified with consistency across participants, supporting the feasibility of context-aware decoding in unconstrained settings.

In this regard, Hyung et al. tackle one of the most persistent limitations of stress detection research: the conflation of workload and stress in task-vs.-rest paradigms. By proposing a rest-vs.-rest design, comparing post-stressor resting EEG with post-relaxation resting EEG, they isolate stress-specific neural dynamics from task engagement effects. Furthermore, their DeepAttNet architecture, built around cross-attention between bilateral ear-EEG channels, demonstrates that subject-independent stress classification can be achieved using only two preauricular electrodes.

An additional axis focuses on state monitoring in operationally realistic tasks, where cognitive states evolve over time and under sustained demands. Zhou et al. address this translational gap by studying cognitive load recognition in simulated flight missions using the Multi-Attribute Task Battery, explicitly moving beyond simplified load elicitation (e.g., arithmetic-only paradigms). By benchmarking end-to-end CNN approaches against more traditional pipelines, they highlight both the promise and the fragility of decoding under prolonged, dynamic task engagement.

The Topic also includes an explicitly translational example of how passive neurophysiological monitoring can support real-time adaptation of interactive environments. Wriessnegger et al. present a proof-of-concept neuroadaptive VR exposure therapy system for spider phobia (VRSpi), integrating VR with real-time monitoring of brain and autonomic responses to automatically adjust stimulus intensity.

Finally, the Topic also covers the system-level implications of translating decoding into adaptive, closed-loop neurotechnology. Jin et al. review EEG-based adaptive closed-loop BCI approaches in neurorehabilitation, emphasizing bidirectional interaction, personalization, and co-adaptation between user and system. Importantly, they highlight practical barriers that become dominant when moving toward real-world use: online vs. offline validation gaps, non-stationarity, transfer learning and calibration demands, user fatigue and compliance, device-level constraints, and the urgent need for standardization in evaluation.

Across the contributions, several overarching themes emerge.

First, multi-dimensional feature integration is becoming a cornerstone of modern pBCI systems. Rather than relying on isolated spatial filters or band-power features, contemporary models increasingly combine connectivity, spectral, temporal, and hierarchical representations. This multi-branch perspective reflects a growing recognition that cognitive and affective states are distributed phenomena, expressed through coordinated neural dynamics rather than localized markers.

Second, subject independence and generalization are central to real-world viability. Calibration-heavy approaches may be acceptable in laboratory research but limit scalability. The adoption of subject-level cross-validation protocols and domain-robust architectures signals a shift toward deployable systems capable of handling inter-individual variability, a critical step for clinical, occupational, and consumer applications.

Third, interpretability is no longer optional. As pBCIs move into contexts involving health, safety, and decision support, transparent reasoning becomes essential. Techniques such as SHAP-based feature attribution, attention-weight analysis, and connectivity-informed graph structures not only improve trustworthiness but also generate neuroscientific insights. In this sense, pBCI research is increasingly bidirectional: it not only applies neuroscience knowledge but also contributes to understanding brain dynamics underlying stress, motor planning, and cognitive control.

Fourth, hardware constraints and algorithmic design are becoming tightly coupled. Ear-EEG systems, sparse montages, and wearable-compatible sensors impose architectural constraints that, in turn, stimulate innovation in lightweight models, cross-attention mechanisms, and compact feature representations. The synergy between device design and algorithmic development is a defining characteristic of the field's maturation.

Moving forward, several open challenges remain. Real-world environments introduce motion artifacts, contextual variability, and multimodal interactions that exceed controlled laboratory conditions. Longitudinal stability, robustness to environmental noise, and integration with other physiological signals will likely define the next frontier of passive BCIs. Ethical considerations, including privacy, informed consent, and responsible AI, will also become increasingly central as continuous brain-state monitoring becomes feasible.

The works presented in this Research Topic collectively demonstrate that passive BCIs are no longer confined to experimental paradigms optimized for controlled settings. Bringing passive BCIs into real-world application requires more than improved classification accuracy. It demands robustness, interpretability, ecological validity, and human-centered design. The contributions gathered here provide compelling evidence that the field is actively addressing these dimensions, moving decisively from laboratory prototypes to practical, deployable neurotechnology.
